# Survey of Traveler's Diarrhea: Epidemiology and Testing Reveal the Source

**DOI:** 10.1155/2019/3569840

**Published:** 2019-11-29

**Authors:** Zhenguo Gao, Muti Mahe, Shabiremu Tuohetamu, Fang Li, Jian Zhang, Yidan Xia, Xiaona Sun, Abuzhalihan Naerkezi, Ruifang Huang, Hongbin Liu, Daxin Ni, Rong Zhang

**Affiliations:** ^1^Xinjiang Uygur Autonomous Region Center for Disease Control and Prevention, Urumqi, China; ^2^China Center for Disease Control and Prevention, Beijing, China

## Abstract

**Objective:**

To understand the causes and transmission routes of, as well as risk factors, for a Salmonella outbreak in a tour group.

**Method:**

A retrospective cohort design was used to conduct an epidemiological field investigation. Real-time fluorescent quantitative PCR, bacterial culture, and serological identification methods were used for pathogen detection and identification.

**Result:**

There were 7 cases of illness, and the attack rate was 46.67%. The onset date was concentrated on May 9 and 10. All cases were found in the tour group, and no cases occurred in the nontour group. The results of this retrospective cohort study showed that the consumption of boiled eggs for breakfast on May 9 was a common factor (*R*^2^ = 6.67, *P*=0.023). *Salmonella enteritidis* was identified from the patients' stool and vomit.

**Conclusion:**

The food poisoning epidemic was caused by *Salmonella enteritidis*. In the summer and autumn, attention should be paid to preservation, processing, and cooking of food to avoid bacterial contamination. To prevent sickness, travelers should know the disease prevalence at their destinations in advance.

## 1. Introduction

*Salmonella* is a Gram-negative bacillus that is highly resistant to the external environment. It can survive for several months in water and soil, and it can survive for 1 to 2 months in feces. It is a common pathogen associated with bacterial food poisoning. *Salmonella enteritidis* is a common serotype of *Salmonella* responsible for food poisoning [1]. Although *Salmonella* food poisoning has been reported, travel-associated food poisoning caused by *Salmonella enteritidis* is rarely documented in domestic disease databases. Epidemiological analysis methods and laboratory tests were used to investigate the outbreak.

## 2. Methods

### 2.1. Identification of the Outbreak

At 10:30 on May 10, 2018, flight CZ6070 arrived in Urumqi. Several patients presented high fever, nausea, vomiting, diarrhea, sore throat, and difficulty breathing on flight CZ6070 from Dubai via Urumqi to Lanzhou. The patients were travelling from a Middle East Respiratory Syndrome (MERS) epidemic area, and some of the patients had respiratory symptoms. According to the literature, fever and gastrointestinal symptoms may be prodromal symptoms in some MERS cases [2–4]. The receiving hospital suspected that the patients were infected with MERS and immediately reported it. After receiving the report, an investigation team was immediately sent to conduct an epidemiological investigation and collect specimens at the Xinjiang Uygur Autonomous Region Center for Disease Control and Prevention. The body temperature of all the passengers on the flight was measured. Medical observations of all the passengers were conducted. The passengers on the flight that did not contain members of the tour group had no fever symptoms. The investigation revealed that the patients were all part of a tour group that had travelled to Dubai and Abu Dhabi. The nontour group did not present disease symptoms. The travel dates were from May 4 to May 10.

The case definition was as follows: passengers on the flight who presented fever plus one additional symptom of nausea, vomiting, abdominal pain, diarrhea, sore throat, or difficulty breathing on or after May 4, 2018.

### 2.2. Epidemiological Investigation

The list of tour group members and hospital records were evaluated, on-the-spot interviews were conducted, and epidemiological questionnaires were completed. We found that 7 people met the inclusion criteria.

### 2.3. Laboratory Testing Methods

Samples from all 7 cases were collected with swabs. Pretreatment excreta and vomit samples were collected. Laboratory tests were performed using real-time PCR, bacterial culture, and serological identification.

### 2.4. Epidemiological Analysis Methods

The analysis was performed as a retrospective cohort study. Epi Info™ (Division of Health Informatics and Surveillance (DHIS), Center for Surveillance, Epidemiology and Laboratory Services (CSELS)) and IBM SPSS Statistic analysis software were used, and the *R*^2^ value was calculated and statistically tested.

## 3. Results

### 3.1. Clinical Features

The main clinical symptoms are shown in Table 1. The temperature of all the fever cases was between 38.5 and 39.0°C. The frequency of vomiting ranged from 2 to 14 times, and the proportion of patients who vomited less than 5 times was 71.43%. The frequency of diarrhea ranged from 2 to 20 times, and the proportion of patients who experienced diarrhea more than 10 times was 66.67%. The main clinical laboratory examination features are shown in Table 2. Most of the patients had elevated neutrophils and decreased lymphocytes. The proportion of people with a sore throat and difficulty breathing was very low.

### 3.2. Time Distribution Characteristics

**Figure 1:**
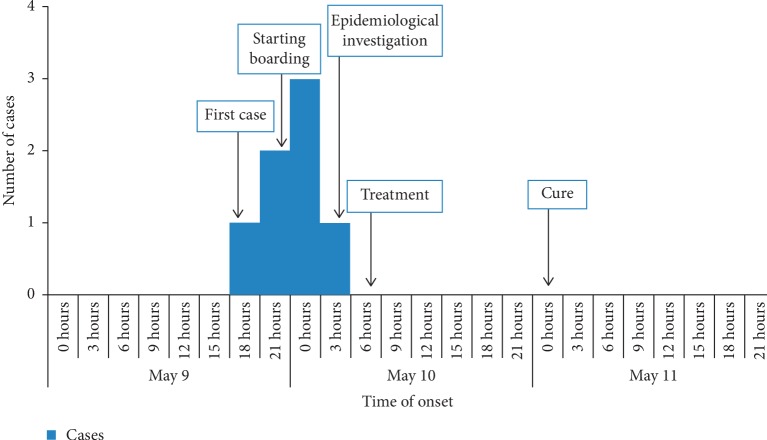
Epidemiological curve of outbreak investigation.

As shown in Figure 1, this outbreak was a typical point-source epidemic. We determined that the outbreak was caused abroad because the first case occurred before boarding.

### 3.3. Population Distribution Characteristics

Seven of the 15 members of the tour group were ill, corresponding to an incidence rate of 46.67%. There were 3 males and 4 females in the affected population. The ages ranged from 5 to 62 years old, and the median age was 46 years old. There was no obvious age distribution. The incidence data showed family aggregation, as 5 patients were from the same family and 2 were in another family. The occupational characteristics of the patients were as follows: 1 student, 2 retirees, 2 employees, 1 civil servant, and 1 unemployed.

### 3.4. Investigation of Respiratory Infections

Dubai and Abu Dhabi belong to the MERS epidemic area. Some of the patients presented respiratory symptoms. Therefore, we conducted an investigation into the exposure to respiratory infections. According to the survey, all 7 patients had no history of contact with camels or bats during the trip. The only animal exposure history was travel to a flamingo park to watch flamingos. However, there was a solid glass window between the visitors and the flamingos. Therefore, there was no chance of direct animal exposure. There was no history of contact with people who had similar symptoms.

### 3.5. Investigation of Intestinal Infectious Disease Exposure

#### 3.5.1. Exposure to Meals

During the trip, the tour group members had only breakfast in common. There was no common exposure history for lunches and dinners for the 7 patients because the tour group did not provide lunch and dinner service. From May 5 to May 8, the tour group members stayed at the same hotel in Abu Dhabi, and the daily breakfast was basically the same. No cases occurred during this time period. Breakfast was served at the Dubai Hotel on May 9, and the meal time was from 10 to 11 a.m. in Dubai time.

#### 3.5.2. Common Exposure to Drinking Water

Mineral water was provided to the tour group daily. Some of the patients did not drink the mineral water and purchased beverages themselves. Some patients brought a cup of hot water from the hotel to drink.

### 3.6. Laboratory Test Results

Throat swabs were collected from all 7 patients. Laboratory testing for common respiratory viruses and respiratory bacteria from the throat swabs was carried out by real-time PCR. The experimental results are shown in Table 3. *Streptococcus pneumoniae*, *Pseudomonas aeruginosa*, and *Haemophilus influenzae* are conditional pathogens. They colonize the upper respiratory tract of normal people. Based on these data and the clinical features, the cause of the outbreak was not these three bacterial species.

**Figure 2:**
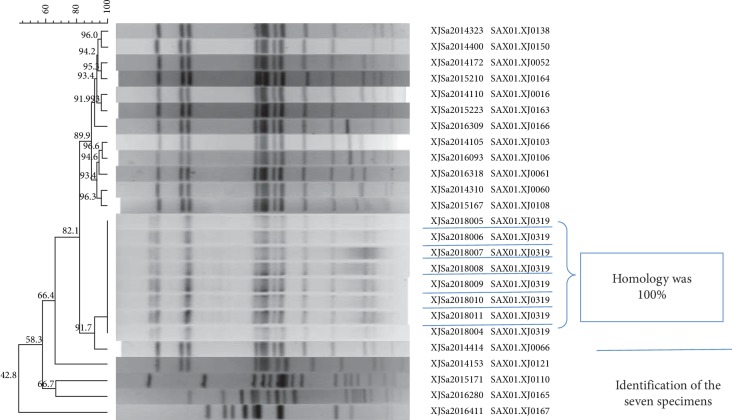
The results of the PFGE experiment.

Stool samples were collected from all 7 patients. Laboratory testing for common pathogens was carried out by real-time PCR. The experimental results are shown in Table 4. After bacterial enrichment culture, strains of *Salmonella* were obtained from stool samples of the 7 patients. After serotype identification, the bacteria were identified as *Salmonella enteritidis*. The results of the PFGE experiment show that the homology to *S. enteritidis* was 100% (Figure 2).

### 3.7. Hypothesis and Verification

#### 3.7.1. Hypothesis

The hypothesis was as follows: the outbreak was caused by eating food contaminated with bacteria during the trip because (1) it was a typical point-source epidemic; (2) the first case occurred in the United Arab Emirates before returning to China; and (3) the common exposure time was from 2 p.m. on May 8 to 1 p.m. on May 9, which is in accordance with the incubation period of *Salmonella enteritidis*. It was speculated that during this period, the common exposure history was related to dinner on the evening of May 8 and breakfast and lunch on May 9.

#### 3.7.2. Verification

During the period of the trip, the tour group members had only breakfast in common. There was no common exposure history for lunch and dinner among the 7 patients because the tour group did not provide lunch and dinner service. A survey of meals showed that breakfast on May 9 was a common factor.

After confirming the suspicious meal, we investigated the breakfast food with a retrospective cohort study on May 9. The results showed that boiled eggs were the most likely risk factor for *S. enteritidis* food poisoning (Table 5). The rate of illness in those exposed to this risk factor was 6.67 times higher than that in those who were not exposed to it.

## 4. Discussion

The main symptoms of Salmonella food poisoning are high fever, abdominal pain, and diarrhea [2, 3]. In contrast to other outbreaks, the proportion of stagnation and vomiting in this outbreak of Salmonella food poisoning was high. The degree of abdominal pain and diarrhea was greater than that of nausea and vomiting.

Fever and gastrointestinal symptoms may be prodromal symptoms in some MERS cases, according to the literature. Although there were few cases of respiratory symptoms in this investigation, we could not rule out MERS before the laboratory results were available. To prevent the spread of MERS in China, we conducted a respiratory epidemiological survey. Exposure to flamingos had no significance for MERS. MERS was excluded by both the epidemiological and laboratory investigations.

According to the literature, food poisoning is caused by eating food contaminated by *Salmonella*. In many papers, the consumption of contaminated eggs, milk, and milk products was the major cause of *Salmonella enteritidis* food poisoning [4–8]. In contrast to hospital admission data analyses that lack full characterization of the nature of the infections, we investigated the epidemiological history in detail [9–11].

The outbreak of food poisoning occurred in the UAE, not in China. Traveler's diarrhea is still the most common travel-associated illness [12, 13]. It was regrettable that we could not travel to the epidemic area to investigate the food-related processes, such as the purchasing, transporting, and cooking, of potentially infected food. If we had been able to travel to the outbreak area, we could have determined whether the food was contaminated, whether the food was not refrigerated during storage, or whether the food was not cooked thoroughly during processing.

Because the epidemic curve of the outbreak showed a typical point-source epidemic, it was possible to infer the possible exposure time by calculating the median time of the disease, the incubation period of the disease and the onset time of the first and last cases, which provides a good opportunity to identify the risk factors.

It can be seen that the members of the tour group were exposed to a similar meal, but other people who ate at the restaurant could not be found, resulting in a limited number of cases and controls. There may also be recall bias by the patients due to the investigation into suspicious foods and the trauma of vomiting and diarrhea.

The outbreak of food poisoning occurred in Dubai, and there are few reports of bacterial food poisoning at this location. When a food poisoning outbreak occurs, it should be stopped in time to prevent further spread. This survey provides a theoretical basis for the prevention and treatment of diarrhea during travelling. Dubai belongs to the Eastern Mediterranean. According to reports in the literature, over 125,000 deaths (3.6% of total deaths) in the Eastern Mediterranean region in 2013 were due to diarrheal diseases, with a greater burden of diarrheal diseases in low- and middle-income countries. Diarrhea-associated deaths per 100,000 children under 5 years of age ranged from one (95% uncertainty interval (UI) = 0-1) in Bahrain and Oman to 471 (95% UI = 245–763) in Somalia [14]. Therefore, diarrhea in the area should be given attention.

Salmonella is one of the leading causes of food-borne enterocolitis worldwide. The main serotypes are *Salmonella typhimurium*, *Salmonella enteritidis*, *Salmonella heidelberg*, and *Salmonella newport* [15]. However, they are different among different countries. Food poisoning caused by *Salmonella* in Germany is the second most common cause, and *Salmonella enteritidis* and *Salmonella typhimurium* account for a high proportion of cases [16, 17]. With the acceleration of globalization, the exchanges between people in various countries have gradually increased, and food poisoning is spreading quickly across borders. To prevent and control infectious diseases, it is important to promote information exchange in the field of infectious diseases globally. This exchange will provide better prospects and hope for the future development of disease prevention and control.

## Figures and Tables

**Table 1:** Clinical features of the patients.

Clinical features	Proportion
Nausea	100
Vomiting	100
Diarrhea	85.71
Stomach ache	85.71
Fever	71.43
Sore throat	28.57
Difficulty breathing	14.28

**Table 2:** The main clinical laboratory examination features of the patients.

	Blood examination results			Fecal examination results		
	Leucocytes(10^9^/L)	Neutrophils (10^9^/L)	Lymphoid (10^9^/L)	Fecal trait	Leucocytes (sample/HP)	Phagocytes (sample/HP)
Case 1	5.31	8.91	0.48↓	Yellow loose stool	3	0
Case 2	10.06↑	9.36↑	0.38↓	Yellow loose stool	0	0
Case 3	13.67↑	12.68↑	0.51↓	Yellow loose stool	0	0
Case 4	12.40↑	11.55↑	0.43↓	Yellow loose stool	26	2
Case 5	13.01↑	11.79↑	1	Yellow loose stool	12	2
Case 6	16.12↑	14.78↑	0.89	Yellow loose stool	5	1
Case 7	22.35↑	21.6↑	0.43↓	Yellow loose stool	1	0

**Table 3:** Common respiratory pathogen test results of the patients' throat swabs.

Respiratory virus species	Test result	Respiratory bacteria	Test result
Parainfluenza virus type 1	Negative	*Mycoplasma pneumoniae*	Negative
Parainfluenza virus type 2	Negative	*Klebsiella pneumoniae*	Negative
Parainfluenza virus type 3	Negative	*Chlamydia pneumoniae*	Negative
Parainfluenza virus type 4	Negative	*Streptococcus pneumoniae*	Positive
Influenza A virus	Negative	*Staphylococcus aureus*	Negative
Influenza B virus	Negative	*Legionella pneumophila*	Negative
Adenovirus	Negative	*Pseudomonas aeruginosa*	Positive
Respiratory syncytial virus type A	Negative	*Moraxella catarrhalis*	Negative
Respiratory syncytial virus type B	Negative	*Bordetella pertussis*	Negative
Enterovirus	Negative	*Haemophilus influenzae*	Positive
Boca virus	Negative	*Mycobacterium tuberculosis*	Negative
Partial lung virus	Negative	*Mycobacterium avium*	Negative
Rhinovirus	Negative	*Acinetobacter baumannii*	Negative
MERS	Negative		
Coronavirus	Negative		

**Table 4:** Pathogen test results of the patients' stool samples.

Pathogen name	Experimental result
*Salmonella*	Positive
*Staphylococcus aureus*	Negative
*Enterobacter sakazakii*	Negative
*Yersinia enterocolitica*	Negative
*Aeromonas hydrophila*	Negative
*Bacillus cereus*	Negative
*Listeria monocytogenes*	Negative
*Escherichia coli* O157	Negative
*Shiga bacillus*	Negative
*Vibrio cholerae*	Negative
*Campylobacter coli*	Negative
*Vibrio parahaemolyticus*	Negative
*Campylobacter jejuni*	Negative

**Table 5:** Retrospective survey of food eaten for breakfast on May 9.

Exposure factors		Cases	Noncases	*R* ^2^	*P* value (Fisher's exact probability method)
Boiled egg	Exposed	5	1	6.67	0.026
	Unexposed	1	7		
Oatmeal	Exposed	3	1	1.88	0.559
	Unexposed	4	6		
Jam	Exposed	4	2	1.78	0.592
	Unexposed	3	5		
Bread	Exposed	2	2	1	1
	Unexposed	5	5		
Cantaloupe	Exposed	5	5	1	1
	Unexposed	2	2		
Fried rice	Exposed	1	1	1	1
	Unexposed	6	6		
Cake	Exposed	1	2	0.61	1
	Unexposed	6	5		
Milk	Exposed	1	3	0.42	0.559
	Unexposed	6	4		
Orange juice	Exposed	1	3	0.42	0.559
	Unexposed	6	4		
Cucumber	Exposed	1	4	0.3	0.266
	Unexposed	6	3		

## Data Availability

The data used to support the findings of this study are included within the article.
